# The ammonia-Slc4a11 axis in T cells alleviates LPS-induced mastitis

**DOI:** 10.3389/fimmu.2025.1537483

**Published:** 2025-04-30

**Authors:** Yuqing Wu, Zhi Li, Peiwen Xi, Yaman Wang, Haowei Guo, Hong Yin, Lei Zhu

**Affiliations:** ^1^ Department of Laboratory Medicine, The First Affiliated Hospital of Nanjing Medical University, Nanjing, China; ^2^ Branch of National Clinical Research Center for Laboratory Medicine, Nanjing, China; ^3^ Department of Breast and Thyroid Surgery, Huai’an First People’s Hospital, Nanjing Medical University, Huai’an, China; ^4^ Health Management Center, The First Affiliated Hospital with Nanjing Medical University, Nanjing, China; ^5^ Department of Breast Surgery, Women’s Hospital of Nanjing Medical University (Nanjing Women and Children’s Healthcare Hospital), Nanjing, China

**Keywords:** ammonia, SLC4A11, mastitis, T cell, LPS

## Abstract

**Background:**

Mastitis is an inflammatory condition of the mammary gland, commonly observed in lactating and non-puerperal women, posing significant health and economic challenges. Lipopolysaccharide (LPS), a component of the outer membrane of Gram-negative bacteria, is a major inducer of mastitis. Ammonia, a key molecule in nitrogen metabolism, has been implicated in inflammatory pathways, yet its specific role in mastitis remains unclear. This study aims to investigate the mechanism by which ammonia influences the development of mastitis, particularly its effects on T cell activity and inflammatory factor expression.

**Methods:**

qRT-PCR and ELISA were performed to measure the levels of IL-6, TNF, and IL-1β in the breast tissue of mice with LPS-induced mastitis, with or without ammonia treatment. HE staining was used to evaluate the degree of inflammation in the mammary tissue. FACS analysis was employed to assess the percentage, viability, and proliferation of immune cells in the breast tissue. CRISPR-Cas9 technology was used to knockout the SLC4A11 gene in T cells.

**Results:**

Ammonia treatment significantly alleviated LPS-induced mastitis by reducing inflammation and inflammatory factor levels. It also decreased the percentage of CD4+ and CD8+ T cells, inhibited T cell viability and proliferation, and reduced pro-inflammatory cytokine expression (TNF and IFN-γ). Knockdown of the ammonia transporter Slc4a11 in T cells exacerbated mastitis, suggesting that Slc4a11 regulates T cell activity and inflammation during the progression of mastitis.

**Conclusion:**

In summary, these findings highlight the critical role of ammonia and its transporter Slc4a11 in LPS-induced mastitis, providing potential therapeutic targets for future interventions.

## 1 Introduction

Mastitis is a prevalent disease characterized by inflammation and damage in mammary glands, imposing substantial economic burdens on the health of women (1). The primary causative factor of mastitis is bacterial infection, with LPS from Gram-negative bacteria such as Escherichia coli playing a pivotal role in initiating the inflammatory response (2). LPS, acting as a potent immune stimulant, triggers a cascade of immune responses when it interacts with toll-like receptor 4 (TLR4) on mammary epithelial cells and immune cells (3). This interaction leads to the activation of nuclear factor kappa B (NF-κB), resulting in the production of pro-inflammatory cytokines such as tumor necrosis factor-alpha (TNF-α) and interleukin-1 beta (IL-1β) (4). The ensuing inflammation causes tissue damage and can lead to a compromised blood-milk barrier, further exacerbating the condition.

Ammonia, a highly reactive nitrogenous compound, is generated through the catabolism of proteins and other nitrogen-containing substances (5). While it is well-known for its ability to exacerbate oxidative stress by promoting the production of reactive oxygen species (ROS) and compromising antioxidant defenses, ammonia may also play regulatory roles in inflammation (6). For instance, low concentrations of ammonia have been shown to enhance the bactericidal activity of neutrophils, which serve as a crucial first line of defense against bacterial infections (7). Moreover, ammonia metabolism may be implicated in the resolution of inflammation, as ammonia can act as a substrate for the production of nitric oxide (NO), a molecule with dual roles in inflammation—exhibiting both pro-inflammatory and anti-inflammatory properties depending on its concentration and context (8).

In terms of immune cell function, ammonia has been shown to influence the activity of various immune cells, such as macrophages, dendritic cells, and T cells (9). For instance, it can impair macrophage phagocytosis and cytokine production, alter dendritic cell maturation and antigen presentation, and suppress T cell proliferation and cytokine secretion (10).

While the direct link between ammonia and mastitis has not been extensively studied, it is plausible to consider the potential impact of ammonia metabolism in the context of mastitis. By targeting the ammonia metabolism pathway, it could be possible to slow down the growth of inflammatory or pathogenic cells within the breast tissue. For instance, inhibiting glutamate dehydrogenase (GDH) activity has been shown to alleviate tumor growth in mouse models of breast cancer, suggesting that similar approaches could be explored for mastitis (11).

In this study, we focus on the role of ammonia in the development of mastitis. By studying the effect of ammonia on the mouse model of mastitis, the mechanism of ammonia in the development of mastitis can be revealed. Our study uses a variety of research methods, including cell separation, flow cytometry analysis, ELISA, qRT-PCR, and CRISPR-Cas9. We showed that ammonia could inhibit LPS-induced mastitis, reduce T cell activity, and decrease the expression of inflammatory factors.

## 2 Methods and materials

### 2.1 Reagents

The list below details the flow cytometry antibodies that were employed (all sourced from BD Biosciences, unless specified otherwise): PE-conjugated antibodies to mouse TNF (MP6-XT22, 554419), Alexa Fluor 700-conjugated antibodies to mouse CD4 (RM4-5, 557956), PerCP-CyTM5.5-conjugated antibodies to mouse CD8α(53-6.7, 551162), FITC-conjugated antibodies to mouse CD45(30-F11, 553079), Fixable Viability Stain 780 (565388), Alexa Fluor 647-conjugated antibodies to mouse IFN-γ(XMG1.2, 557735), DNasel (Sigma-Aldrich, DN25), Collagenase,type IV(Sigma-Aldrich, C5138), RBC lysis buffer(beyotime, C3702), NH4Cl(Sigma-Aldrich, 213330).

### 2.2 Mice model

The 6 weeks-old wild-type C57BL/6 mice were purchased from GemPharmatech Co Ltd (Nanjing, China). The mice were housed in a controlled barrier environment, and all experimental procedures were executed in strict compliance with the ethical standards set by the Ethical Review Committee for Laboratory Animal Welfare at Nanjing Medical University. Throughout the study, the mice were maintained under a 12h light/dark cycle at a temperature range of 22-24°C, with ad libitum access to food and water.

For the induction of mastitis, Eight-week-old C57BL/6 mice were injected LPS directly into the fourth pair of mammary gland tissues. After injection, the mice were divided into two treatment groups: a control group receiving a vehicle (Vehicle) and an experimental group treated with ammonium chloride (NH4Cl, referred to as Ammonia). At 72 hours following the administration of anesthesia with isoflurane, the mice underwent retro-orbital blood sampling (100ul) and sacrificed for mastitis tissues under sterile operating conditions for subsequent processing.

### 2.3 Magnetic-activated cell sorting cell separation

For isolation of mouse T cells from spleen, CD3^+^ cells were enriched from spleens by using a CD3 MicroBead Kit, according to the manufacturer’s instructions (Miltenyi Biotec). Enriched CD3^+^ cells were stained with CD3 antibodies and detected by the flow cytometer. The purity of T cells was > 95%.

### 2.4 H&E staining

Mammary gland samples from mice were submerged in a 10% phosphate-buffered formalin solution for overnight fixation. Subsequently, the samples underwent a dehydration process prior to being embedded in paraffin at 58°C for an extended period. Once solidified, the paraffin-embedded tissues were sectioned into 5–10 μm thick slices. These sections were rehydrated through a series of ethanol washes and stained with a hematoxylin solution composed of 0.1% hematoxylin, 5% potassium sulfate (Kal [SO4]2), and 0.02% potassium iodate (KIO3). Following the primary staining, the sections were counterstained with a 1% eosin solution. Dehydration and clearing were performed using xylene, and the slides were mounted with a neutral gum medium. Imaging of the H&E-stained sections was conducted using a Motic slide scanner (Motic, EasyScan NFC, Xiamen).

### 2.5 Enzyme-linked immunosorbent assay

C57BL/6 mice at eight weeks of age received an *in situ* injection of LPS into the fourth pair of mammary gland tissues. After 24 hours, the mice were anesthetized using isoflurane, and 100 microliters of blood were collected via the retro-orbital route. The blood samples were then centrifuged at 3000 revolutions per minute (rpm) for 5 minutes to separate plasma. The concentrations of IL-6, TNF-α, and IL-1β in the plasma were quantified using specific ELISA kits (R & D systems), following the manufacturer’s guidelines.

### 2.6 Flow cytometry analysis

To isolate immune cells associated with inflammation, the mice were humanely euthanized, and their mammary glands were extracted and minced into small pieces. These pieces were enzymatically digested in RPMI 1640 medium (Gibco) containing 1 mg/ml collagenase type IV and 0.1 mg/ml DNase I for 2 hours in a 37°C shaking incubator at 200 revolutions per minute (r.p.m.). The resulting cell suspension was centrifuged over a Percoll density gradient (GE Healthcare) to separate mononuclear cells at the 40% to 80% interface. These cells were further processed by filtration through a 70 μm cell strainer. Spleens were also processed by crushing through a 40 μm cell strainer, followed by red blood cell lysis using an RBC lysis buffer. For intracellular cytokine staining, cells were stimulated with 50 ng/ml phorbol 12-myristate 13-acetate (PMA, Sigma-Aldrich), 1 μg/ml ionomycin (Sigma-Aldrich), and 2.5 μg/ml monensin at 37°C in RPMI 1640 medium supplemented with 10% fetal bovine serum (FBS) for 4 hours. Cells were then labeled with surface antibodies, fixed with a Fixation/Permeabilization buffer for 40 minutes, and subsequently stained with intracellular antibodies.

### 2.7 Quantitative real-time polymerase chain reaction

Total RNA was extracted from mouse mammary tissues using TRIzol reagent (Invitrogen) and reverse transcribed into complementary DNA (cDNA) using HiScript II Q Select RT SuperMix for qPCR (Vazyme, Cat: R232-01). qRT-PCR was performed on the ABI 7500 Thermocycler (Applied Biosystems) in 20 μl reaction volumes, which included the cDNA, specific primers, and AceQ qPCR SYBR Green Master Mix (Vazyme, Cat: Q121-02).

### 2.8 CRISPR-Cas 9

Cells were seeded at a density of 105 cells/well in a 24-well plate one day prior to transfection. Two 1.5 mL EP centrifuge tubes, labeled as tube A and tube B, were prepared. In tube A, 25 µL of Opti-MEM (Cat# 11095080, Thermo Fisher), 36 pmol of Cas9 protein (Cat# Z03469, GenScript), and 36 pmol of EasyEditsgRNA were combined and incubated for 15 min at room temperature. Meanwhile, tube B received 25 µL of Opti-MEM and 1.5 µL of Lipofectamine 3000 (Cat# L3000015, Thermo Fisher), mixed gently, and incubated at room temperature for 10 min. After incubation, the contents of tubes A and B were gently combined and incubated at room temperature for an additional 10 min. The resulting transfection mixture (50 µL per well) was added to the cells in the 24-well plate and incubated at 37°C. Cells were harvested 72 h post-transfection for subsequent processing.

### 2.9 Adoptive transfer

T cells were purified from spleen using T cell isolation kit (Miltenyi Biotec, Cat# 130-094-973). Then we employed CRISPR-Cas9 technology to specifically knockout the SLC4A11 gene in T cells. The 1*10^6^ gRNA-Ctrl and gRNA-Slc4a11 T cells in the presence of anti-CD3 Ab (2 μg/mL) and anti-CD28 Ab (5 μg/mL) were respectively intravenous injected into *Rag1^-/-^* mice, followed by construction of mastitis model.

### 2.10 Activation of T cell

The 1×10^5^ gRNA-Ctrl and gRNA-Slc4a11 T cells were incubated in 96-well plate. T cells were activated with anti-CD3 Ab (2 μg/mL) and anti-CD28 Ab (5 μg/mL) in RPMI 1640 included murine IL-2(5ng/ml).

### 2.11 Statistical analysis

The data were analyzed by GraphPad Prism 10.0 software and are presented as the mean ± SEM. The statistics were analyzed by using an unpaired t test for two groups and one-way ANOVA for multiple groups. P values were provided as *, P < 0.05; **, P < 0.01; and ***, P < 0.001.

## 3 Results

### 3.1 Ammonia inhibits LPS-induced mastitis

**Figure 1:**
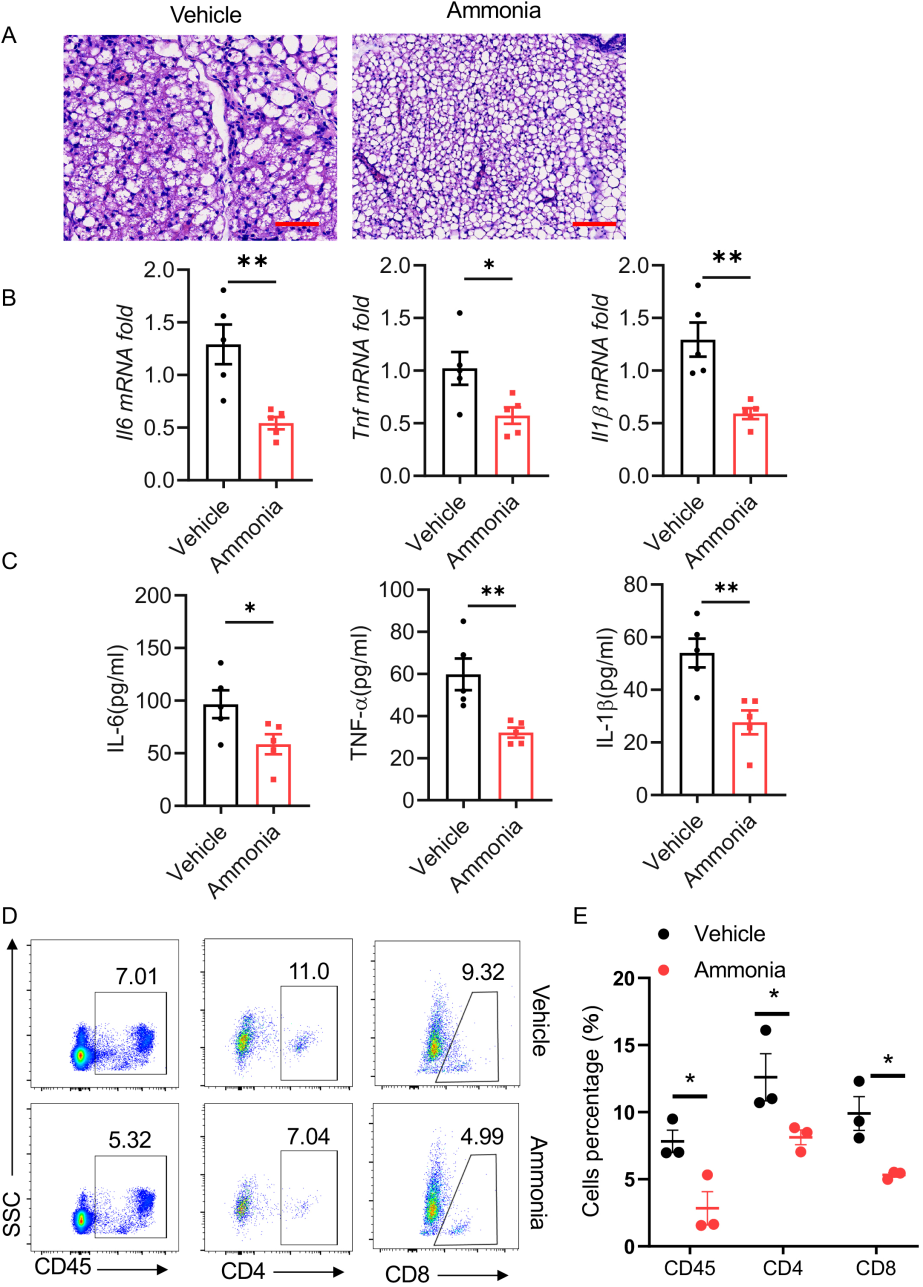
Ammonia inhibits LPS-induced mastitis. **(A)** Histological analysis of breast tissue stained with H&E. The left panel shows the vehicle control group, and the right panel represents the ammonia treatment group (Scale bar: 50 μm). **(B)** Quantitative analysis of mRNA expression levels of inflammatory cytokines (Il6, Tnf, Il1β) in breast tissue. **(C)** Concentrations of IL-6, TNF-α, and IL-1β in serums measured by ELISA. **(D)** Flow cytometric analysis of CD45^+^ immune cells, CD4^+^ T cells and CD8^+^ T cells in breast tissue. **(E)** The percentages of CD45+ immune cells, CD4^+^ T cells and CD8^+^ T cells in the control group and ammonia treatment group. Data are presented as mean ± SEM. *p < 0.05, **p < 0.01.

To investigate the role of ammonia in the development of mastitis, we first established a mouse mastitis model by administering LPS (0.1 mg/ml). Histological examination via hematoxylin and eosin (HE) staining revealed that, compared to the control group, ammonia treatment significantly alleviated inflammation in the mammary glands of mice (**Figure 1A**). Further analysis using quantitative PCR (qPCR) demonstrated that the mRNA expression levels of key inflammatory factors, including TNF-α, IL-6, and IL-1β, were significantly reduced in the ammonia treatment group (**Figure 1B**). Consistent with these findings, enzyme-linked immunosorbent assay (ELISA) experiments showed that the concentrations of TNF-α, IL-6, and IL-1β were also markedly decreased in the ammonia treatment group (**Figure 1C**). Additionally, flow cytometry analysis revealed that the percentages of CD45-positive immune cells, as well as CD4-positive and CD8-positive T cells, were significantly lower in the ammonia treatment group (**Figures 1D, E**).

In summary, our findings demonstrate that ammonia treatment effectively mitigates LPS-induced inflammation and modulates the immune cell composition within breast tissue.

### 3.2 Ammonia reduces T cell viability in LPS-induced mastitis

**Figure 2:**
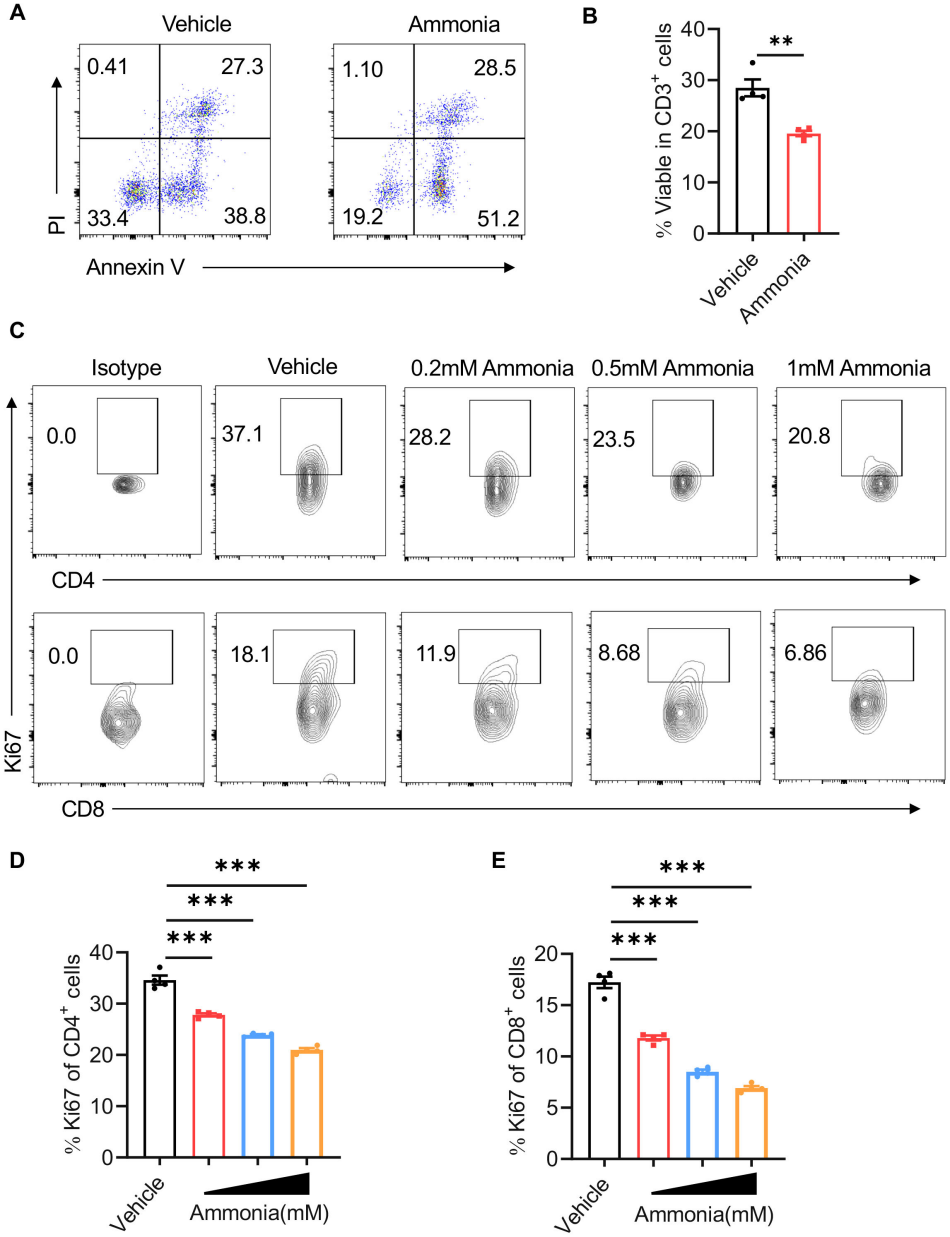
Ammonia reduces T cell viability in LPS-induced mastitis. **(A)** Flow cytometric analysis of PI+ Annexin V+ of CD3^+^ cells in breast tissue. **(B)** The percentages of viable CD3^+^ cells. **(C)** Flow cytometric analysis of CD4^+^ Ki67^+^ and CD8^+^ Ki67^+^ T cells at different concentrations of ammonia (0.2mM, 0.5mM, 1mM). **(D)** Quantification of Ki67^+^ cells within CD4^+^ T cells. **(E)** Quantification of Ki67^+^ cells within CD8^+^ T cells. Data are presented as mean ± SEM. **p < 0.01, ***<0.001.

To further elucidate the impact of ammonia on T cell activity in mastitis, we conducted flow cytometric analyses on mammary tissue samples from two distinct groups. The findings indicated that the percentage of T cell death was significantly elevated in the ammonia treatment group relative to the control group (**Figures 2A, B**). This observation implies a substantial dampening of T cell activity, which is indicative of an anti-inflammatory effect. We then extended our analysis to separately examine the activity of CD4-positive and CD8-positive T cells using flow cytometry. The results demonstrated that ammonia treatment led to a significant decrease in the proliferation of both CD4-positive and CD8-positive T cells (**Figure 2C**). This reduction in T cell proliferation was accompanied by alleviation of inflammation and exhibited a clear dose-dependent relationship (**Figures 2D, E**).

Collectively, these findings indicate that ammonia treatment significantly diminishes the viability and proliferation of both CD4^+^ and CD8^+^ T cells in LPS-induced mastitis, thereby contributing to the observed anti-inflammatory effects.

### 3.3 Ammonia reduced LPS-induced mastitis through T cells

**Figure 3:**
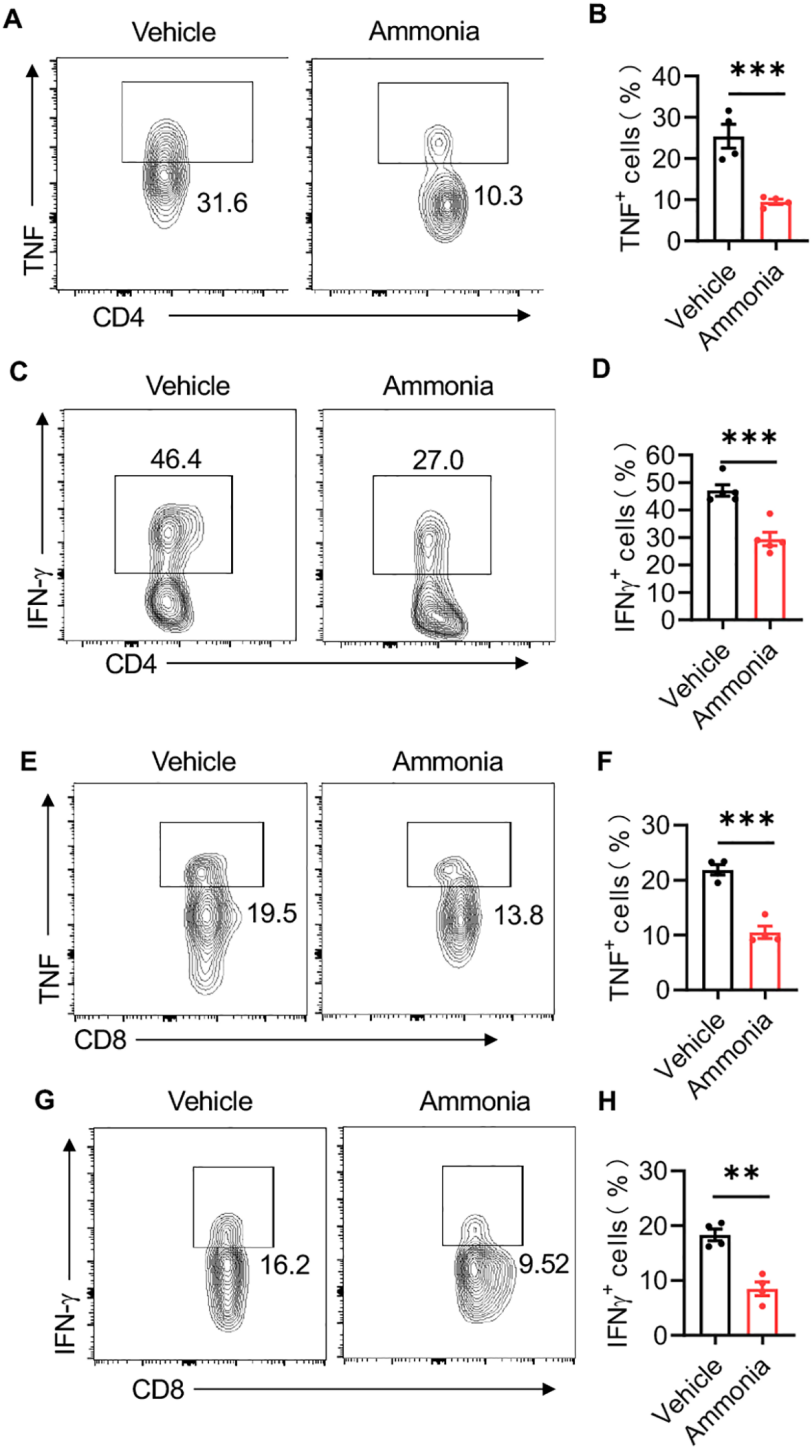
Ammonia reduces LPS-induced mastitis through T cells. **(A, C)** Flow cytometric analysis of TNF^+^ and IFNγ^+^ cells within CD4^+^ T cells in breast tissue. **(B, D)** The percentages of TNF^+^ and IFNγ^+^ cells within CD4^+^ T cells in the control group and ammonia treatment group. **(E, G)** Flow cytometric analysis of TNF^+^ and IFNγ^+^ cells within CD8^+^ T cells. **(F, H)** The percentages of TNF^+^ and IFNγ^+^ cells within CD8^+^ T cells in the control group and ammonia treatment group. Data are presented as mean ± SEM. **p < 0.01, ***<0.001.

Recent studies have highlighted that the accumulation of ammonia is a critical mechanism underlying the impaired pro-inflammatory function of effector T cells (12). In our investigation into the role of T cells in the anti-inflammatory effects of ammonia on mastitis, we observed that, compared to the control group, the activity of CD4-positive T cells was significantly diminished in the ammonia-treated group. This was accompanied by marked reductions in the expression levels of key inflammatory markers, including TNF and IFNγ (**Figures 3A-D**). Similarly, ammonia treatment also led to a notable decline in the activity of CD8-positive T cells, with a corresponding decrease in the expression of their related inflammatory indicators (**Figures 3E-H**).

Overall, the results demonstrate that ammonia treatment significantly inhibits the activation of CD4^+^ and CD8^+^ T cells, as shown by the decreased proportions of TNF^+^ and IFNγ^+^ cells. This highlights the potential of ammonia as an anti-inflammatory mediator in LPS-induced mastitis.

### 3.4 Downregulated the expression of Slc4a11 promotes LPS-induced mastitis

**Figure 4:**
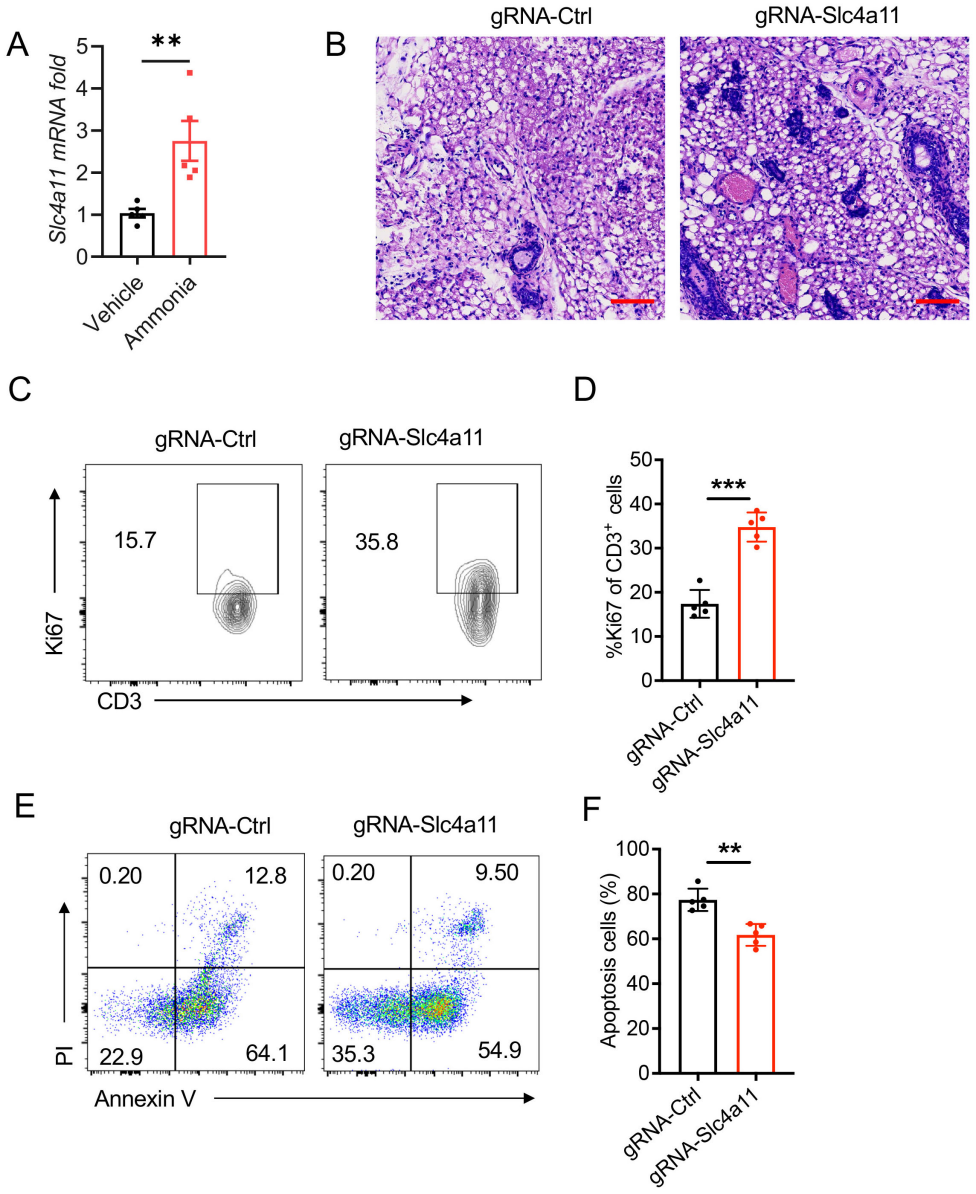
Downregulated the expression of Slc4a11 promotes LPS-induced mastitis. **(A)** Quantitative analysis of Slc4a11 mRNA expression levels in T cells. **(B)** Histological analysis of breast tissue stained with H&E (Scale bar: 50 μm). The left panel displays the control group treated with gRNA-Ctrl and the right panel shows the group treated with gRNA targeting Slc4a11 (gRNA-Slc4a11). **(C, D)** Flow cytometric analysis of CD3^+^ Ki67^+^ T cells in the gRNA-Slc4a11 group compared to the gRNA-Ctrl group. **(E, F)** The percentages of apoptosis cells in the gRNA-Slc4a11 group compared to the gRNA-Ctrl group. Data are presented as mean ± SEM. **p < 0.01, ***<0.001.

To elucidate the potential mechanisms underlying ammonia’s ability to suppress mastitis progression, we focused on the ammonia transport-related gene SLC4A11. We then validated this hypothesis through quantitative PCR (qPCR) analysis. The results revealed that the mRNA expression level of SLC4A11 was significantly upregulated in the ammonia treatment group compared to the control group (**Figure 4A**).

Subsequently, we employed CRISPR-Cas9 technology to specifically knockout the SLC4A11 gene in T cells. These modified T cells were then adoptively transferred into the mammary tissue. HE staining demonstrated that deletion of SLC4A11 in T cells led to a significant exacerbation of mastitis severity (**Figure 4B**). Furthermore, the knockout of the Slc4a11 gene leads to an increased proportion of T cells expressing Ki67 (**Figures 4C, D**) and increased percentages of living cells (**Figures 4E, F**) after the adoptive transfer.

### 3.5 Downregulated the expression of Slc4a11 promotes LPS-induced mastitis through T cells

**Figure 5:**
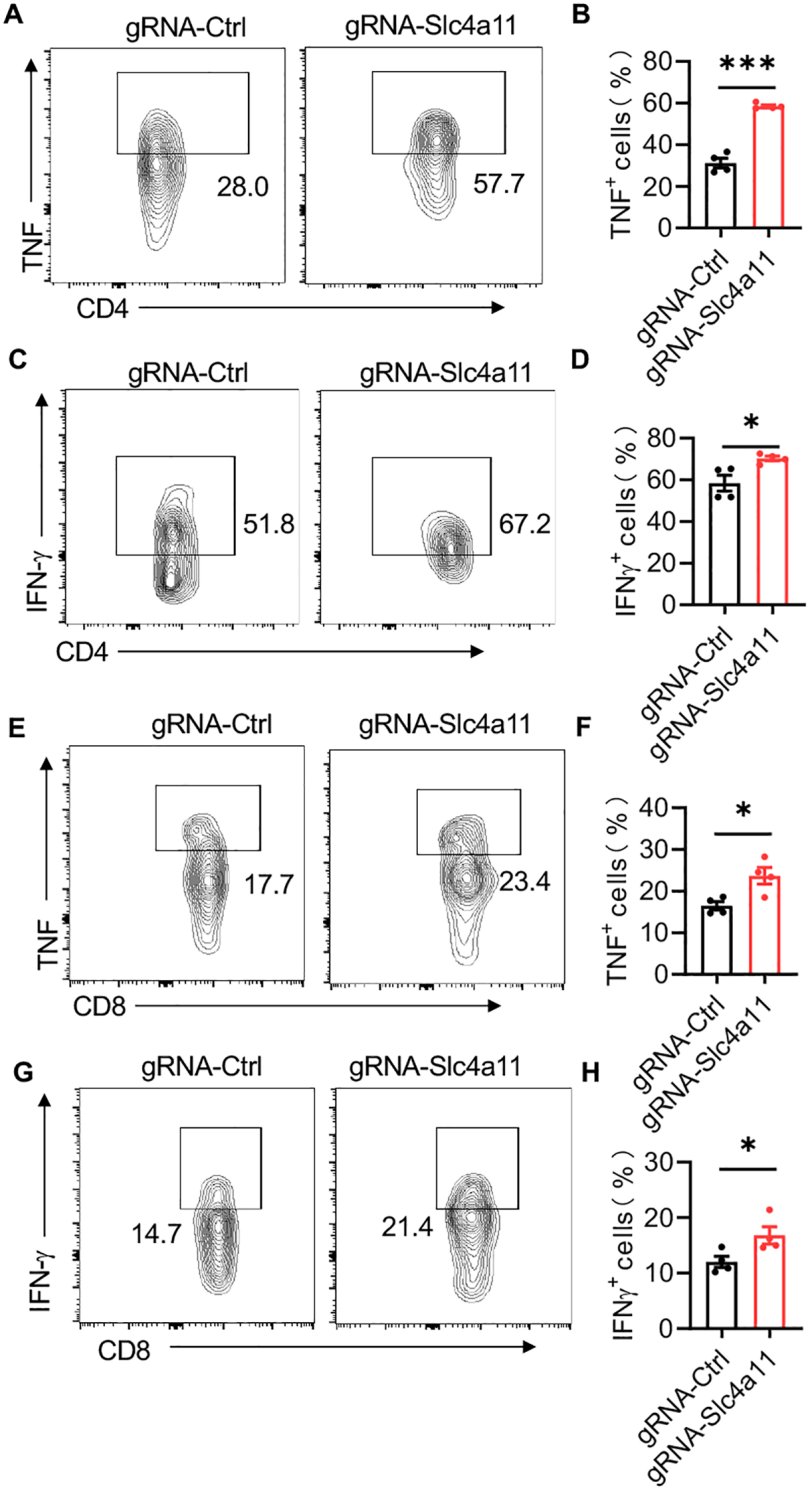
Slc4a11 Downregulation Enhances T Cell-Mediated Inflammation in LPS-Induced Mastitis. **(A, C)** Flow cytometric analysis of TNF^+^ and IFNγ^+^ cells within CD4^+^ T cells. **(B, D)** The percentages of TNF^+^ and IFNγ^+^ cells within CD4^+^ T cells in the gRNA-Slc4a11 group compared to the gRNA-Ctrl group. **(E, G)** Flow cytometric analysis of TNF^+^ and IFNγ^+^ cells within CD8^+^ T cells. **(F, H)** The percentages of TNF^+^ and IFNγ^+^ cells within CD8^+^ T cells in the gRNA-Slc4a11 group compared to the gRNA-Ctrl group. Data are presented as mean ± SEM. *p < 0.05, ***<0.001.

To determine whether Slc4a11 regulates the progression of mastitis through modulation of T cells, we employed CRISPR-Cas9 technology to knock down Slc4a11 expression specifically in T cells. Compared to the control group, the knockdown of Slc4a11 expression resulted in a significant increase in the expression levels of key inflammatory markers, including TNF and IFNγ (**Figures 5A-D**). Similarly, in CD8-positive T cells, the expression levels of TNF and IFNγ were also markedly elevated following Slc4a11 knockdown (**Figures 5E-H**).

These findings suggest that downregulation of Slc4a11 enhances the activation of both CD4^+^ and CD8^+^ T cells, as evidenced by the increased proportions of TNF^+^ and IFNγ^+^ cells. This indicates that Slc4a11 plays a crucial role in modulating T cell responses and thereby influencing the inflammatory process in LPS-induced mastitis.

## 4 Discussion

Our study provides evidence that ammonia plays a significant role in modulating T cell activity and thereby influencing the progression of mastitis. This aligns with recent research showing that SLC4A11 mediates conductive H+(OH-) transport, which is stimulated by raising the extracellular pH (pHe); similarly, ammonia-induced whole cell currents were also stimulated by an increase in pHe (13–15). This suggests that SLC4A11 could be a key player in the modulation of T cell activity in mastitis.

SLC4A11, a member of the SLC4 family of transporters, is known for its role in transporting dicarboxylate and bicarbonate anions (16, 17). In the context of mastitis—an inflammatory condition of the mammary glands—SLC4A11 may contribute to the pathogenesis by influencing the transport of these ions, thereby affecting cellular pH and potentially modulating the inflammatory response. The precise mechanisms by which SLC4A11 operates in mastitis are not fully understood but likely involve multiple pathways. One possibility is that SLC4A11 helps maintain cellular homeostasis by regulating the transport of ammonia, a byproduct of protein metabolism that can be toxic at high concentrations (18). In conditions like mastitis, where inflammation is heightened, the role of SLC4A11 in managing ammonia levels could be particularly crucial.

Furthermore, SLC4A11 has been implicated in the regulation of cellular energy metabolism and may influence ATP production in mitochondria (19, 20). This function could be highly relevant in mastitis, as cellular energy metabolism is often altered during inflammation and may affect the ability of cells to respond to inflammatory stimuli (21). Additionally, SLC4A11 has been identified as a candidate gene associated with resistance to mastitis in dairy cows (22), suggesting that genetic variations in this gene could influence an animal’s susceptibility to the disease.

The modulation of ammonia levels and the targeting of Slc4a11 offer a potential therapeutic strategy for the treatment of mastitis. Recent studies suggest that periductal mastitis is an inflammatory disease related to bacterial infection and consequent immune responses, which supports the idea that targeting the immune response, such as T cell activity, could be beneficial (23–25). Our study adds to this body of work by demonstrating that ammonia, through its interaction with Slc4a11, can inhibit the activity of pro-inflammatory T cells, providing a means to dampen the inflammatory response and protect against tissue damage associated with mastitis.

While our study provides novel insights into the role of ammonia and Slc4a11 in mastitis, there are limitations that warrant further investigation. The controversy surrounding the exact mode of ammonia transport by SLC4A11 necessitates further research (20, 26, 27). Future studies should aim to validate our findings in clinical samples and investigate the detailed molecular mechanisms by which ammonia and Slc4A11 interact to regulate T cell activity and inflammation. Additionally, understanding the long-term effects of ammonia treatment on immune function and mastitis recurrence will be crucial for the development of safe and effective therapeutic strategies.

## 5 Conclusion

In conclusion, our study sheds light on the intricate relationship between ammonia, T cell activity, and mastitis progression. The findings suggest that targeting the ammonia-Slc4a11 axis may offer a promising approach for the management of mastitis, warranting further research into its therapeutic potential. By building on the recent literature, we can better understand the complex interactions at play and develop more targeted treatments for mastitis.

## Data Availability

The raw data supporting the conclusions of this article will be made available by the authors, without undue reservation.
